# Intracellular Ca^2+^ dynamics in the ALA neuron reflect sleep pressure and regulate sleep in *Caenorhabditis elegans*

**DOI:** 10.1016/j.isci.2022.104452

**Published:** 2022-05-23

**Authors:** Shinichi Miyazaki, Taizo Kawano, Masashi Yanagisawa, Yu Hayashi

**Affiliations:** 1 International Institute for Integrative Sleep Medicine (WPI-IIIS), University of Tsukuba, Tsukuba, Ibaraki 305-8575, Japan; 2 PhD Program in Humanics, School of Integrative and Global Majors, University of Tsukuba, Tsukuba, Ibaraki 305-8575, Japan; 3 Department of Molecular Genetics, University of Texas Southwestern Medical Center, Dallas, TX 75390, USA; 4 Life Science Center for Survival Dynamics (TARA), University of Tsukuba, Tsukuba, Ibaraki 305-8577, Japan; 5 R&D Center for Frontiers of Mirai in Policy and Technology (F-MIRAI), University of Tsukuba, Tsukuba, Ibaraki 305-8575, Japan; 6 Department of Human Health Sciences, Graduate School of Medicine, Kyoto University, Sakyo-ku, Kyoto 603-8363, Japan; 7 Department of Biological Sciences, Graduate School of Science, University of Tokyo, Bunkyo-ku, Tokyo 113-0033, Japan

**Keywords:** Neuroscience, Behavioral neuroscience, Cellular neuroscience

## Abstract

The mechanisms underlying sleep homeostasis are poorly understood. The nematode *Caenorhabditis elegans* exhibits 2 types of sleep: lethargus, or developmentally timed, and stress-induced sleep. Lethargus is characterized by alternating cycles of sleep and motion bouts. Sleep bouts are homeostatically regulated, i.e., prolonged active bouts lead to prolonged sleep bouts. Here we reveal that the interneuron ALA is crucial for homeostatic regulation during lethargus. Intracellular Ca^2+^ in ALA gradually increased during active bouts and rapidly decayed upon transitions to sleep bouts. Longer active bouts were accompanied by higher intracellular Ca^2+^ peaks. Optogenetic activation of ALA during active bouts caused transitions to sleep bouts. Dysfunction of CEH-17, which is an LIM homeodomain transcription factor selectively expressed in ALA, impaired the characteristic patterns of ALA intracellular Ca^2+^ and abolished the homeostatic regulation of sleep bouts. These findings indicate that ALA encodes sleep pressure and contributes to sleep homeostasis.

## Highlights

• ALA gradually increases its activity during motion bouts during lethargus in *C. elegans*
• Dysfunction or artificial activation of ALA perturbs the sleep structure
• ALA plays a crucial role in homeostatic sleep regulation

## Graphical abstract

**Figure:**
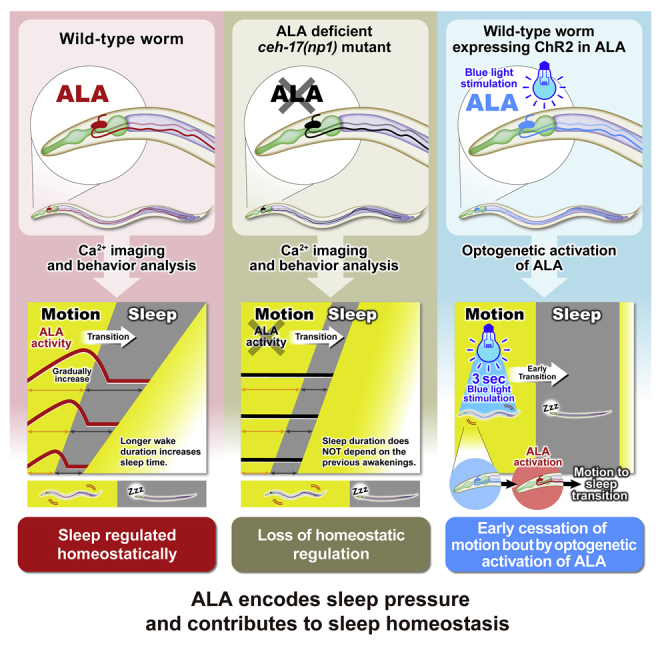

## Introduction

Sleep is a widely conserved state that serves diverse roles (Anafi et al., 2019; Miyazaki et al., 2017). In addition to the circadian clock, sleep is under the control of homeostatic regulation, i.e., prolonged wakefulness leads to increased sleep pressure (Achermann, 2004; Borbély, 1982). Thus, the neurons that govern the switch between vigilance states are likely regulated by a system that somehow encodes the preceding amount of wakefulness as neuronal activity. Identifying the neuronal substrate of this homeostatic system is expected to largely contribute to our understanding of sleep regulation.

Neurons whose activity represents accumulated sleep pressure and promotes sleep drive have been identified. In fruit flies, a subset of neurons in the ellipsoid body exhibits an increased firing rate and cytosolic Ca^2+^ following prolonged wakefulness, which promotes entry into sleep (Liu et al., 2016). Similarly, in mice, neurons in the zona incerta expressing Lhx6 and neurons in the brainstem expressing neurotensin exhibit increased Fos expression under prolonged wakefulness and promote non-rapid-eye movement (REM) sleep (Kashiwagi et al., 2020; Liu et al., 2017). In all cases, the prior history of wakefulness is somehow encoded as neuronal activity. The underlying circuitry or molecular mechanisms, however, remain largely unknown.

Homeostatic regulation is also crucial for shaping the sleep architecture. Rodents cycle between non-REM sleep and REM sleep, and an optimal sleep structure is thought to be crucial for maintaining brain temperature and slow wave activity (Deboer et al., 1994; Hayashi et al., 2015). The duration of a non-REM sleep episode positively correlates with the duration of the prior REM sleep episode (Benington and Heller, 1994). GABAergic neurons in the midbrain ventrolateral periaqueductal gray, which strongly inhibit REM sleep (Hayashi et al., 2015; Weber et al., 2015), exhibit activity that positively correlates with the duration of the preceding REM sleep episode, and their activity gradually decreases during non-REM sleep (Weber et al., 2018). This characteristic activity pattern explains how the alternation between 2 types of sleep can be homeostatically regulated but does not clarify the underlying cellular or molecular mechanisms.

The nematode *Caenorhabditis elegans* is an effective model animal for understanding behavior and physiology at multiscale levels from molecular and cellular to circuit levels. Lethargus in nematodes is a state that precedes molting and is characterized by repeated entrance into an inactive locomotor state (sleep bout) (Cassada and Russell, 1975; Huang et al., 2018; Iwanir et al., 2013; Raizen et al., 2008). Sleep bouts during lethargus satisfy the criterion for sleep, including elevated arousal threshold, behavioral quiescence that can be rapidly reversed to an active state, and homeostatic regulation (Campbell and Tobler, 1984; Raizen et al., 2008). Genetic studies have identified common molecular mechanisms underlying sleep regulation in nematodes and mammals (Funato et al., 2016; Hu et al., 2020; Nakai et al., 2020; Singh et al., 2014; Turek et al., 2013). Homeostatic systems are thought to underlie sleep regulation in nematodes as well as mammals. Sensory stimuli cause a transition to an active locomotor state, which is followed by prolonged rebound sleep bouts (Nagy et al., 2014; Raizen et al., 2008). Even without any perturbation, sleep bouts are alternated with naturally occurring active locomotor states (motion bouts), and the patterns of alternation between these 2 states comprise the sleep architecture in nematodes. Similar to arousal induced by sensory stimuli, naturally occurring motion bouts trigger homeostatic responses, i.e., longer motion bouts are followed by longer sleep bouts (Iwanir et al., 2013; Nagy et al., 2014). These homeostatic properties of sleep bouts suggest that some neuron(s) accumulate information about prior sleep/wake histories and encode the pressure to enter quiescence. To date, however, there are no reports of such neurons.

In the present study, we performed multi-neuronal Ca^2+^ imaging in nematodes during lethargus and observed that Ca^2+^ activity in a neuron termed ALA highly correlates with the prior motion bout. The detailed properties and function of ALA activity were therefore assessed.

## Results

### ALA is activated upon motion bouts during lethargus

**Figure 1:**
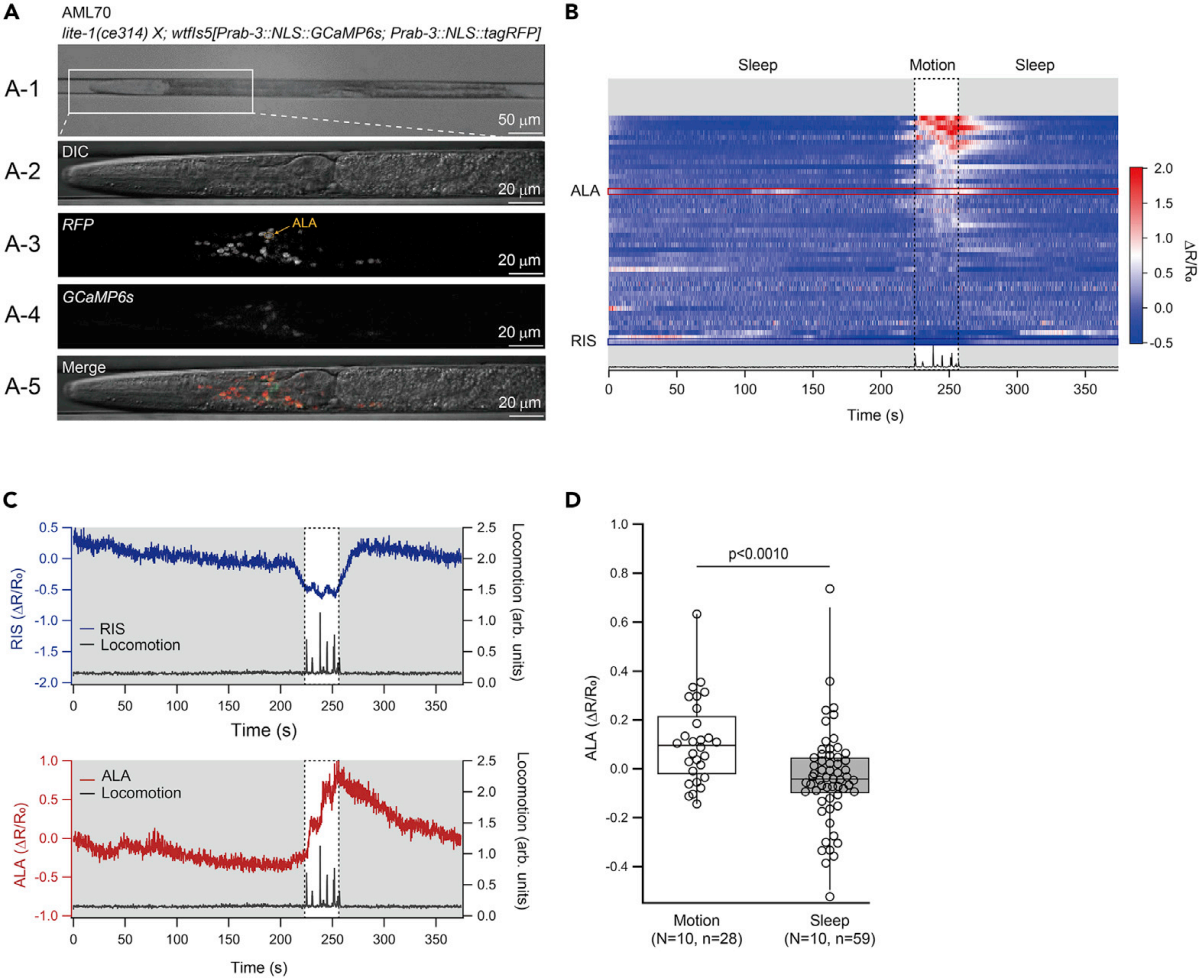
Ca^2+^ imaging in multiple neurons revealed that ALA is activated during motion bouts (A) Microfluidic chamber (Worm Sheet) for Ca2+ imaging (A-1), magnified image of a mounted worm (A-2), and fluorescence signals detected from the head of a mounted worm (A-3, 4, 5) are shown. (B) Representative data from 1 individual showing the changes in GCaMP6s signals. Each row represents a single neuron. Neurons were lined up in order based on Ca^2+^ activity during the motion bout (dotted rectangle). Gray areas represent sleep bouts. The bottom panel shows the locomotion of the animal. The red and blue rectangle shows the signal from ALA and RIS, respectively. (C) Patterns of Ca^2+^ activity of RIS and ALA. The same data as (B) are shown. The gray area represents sleep bouts, and the dotted rectangle indicates a motion bout. (D) Comparison of averaged ALA activity during motion and sleep bouts. p-value in Mann-Whitney’s U test is indicated. N and n in the figure represent numbers of animals and bouts, respectively.

We conducted multi-neuronal Ca^2+^ imaging using a transgenic strain that expresses GCaMP6s under the *rab-3* promoter. In this strain, GCaMP6s is localized to the nuclei, allowing for the segregation of signals derived from neighboring neurons. Animals were placed in a custom-made thin microfluidic chamber to prevent large movements (Figure 1A). We assigned the state of each animal as either in a sleep bout or in a motion bout based on the following criteria; a sleep bout was defined as a series of time points classified as nonlocomotor active that lasts longer than 6 s. The remaining series of time points were classified as motion bouts (see STAR Methods). Under this condition, during lethargus, the animals repeatedly entered sleep bouts and motion bouts as previously described (Iwanir et al., 2013; Nagy et al., 2014; Raizen et al., 2008). L4 larvae that were paralyzed by treatment with sodium azide never entered motion bouts and thus exhibited a high fraction of quiescence (Figures S1A and S1B), confirming that the motion bouts detected in our condition are not the result of involuntary movements due to changes in focus or passive drift. In addition, a previous study revealed that worms placed in microfluidic chambers frequently enter sleep-like states (Gonzales et al., 2019). To assess whether the sleep bouts observed in our imaging condition are a result of entrance to lethargus or due to placing the worms in the microfluidic chamber, we also observed L4 larvae that have not entered lethargus. As a result, these L4 larvae never entered sleep bouts and thus exhibited a low fraction of quiescence (Figure S1A), indicating that the sleep bouts observed in our study are not a result of placing the worms in a microfluidic chamber. Perhaps microfluidic-induced sleep was not observed in our study because imaging was performed within 15 min of loading worms into the microfluidic chamber, whereas the latency to enter microfluidic-induced sleep typically exceeds 10 min (Gonzales et al., 2019), and also because of the differences in the design of the microfluidic chambers.

Overall, the Ca^2+^ activity in the neurons appeared very low during lethargus, consistent with previous reports (Nichols et al., 2017) (Figure 1B; Video S1). Some neurons (31% of the recorded neurons) exhibited increased Ca^2+^ activity during motion bouts, i.e., the average ΔR/R_0_ during motion bouts exceeded the average ΔR/R_0_ across the whole recording session by 20% (Figure 1B). The remaining neurons (69% of the recorded neurons) did not exhibit increased Ca^2+^ activity, even when the animal entered a motion bout (Figure 1B).

Video S1. Representative data of Ca2+ imaging of RIS and ALA during lethargus, related to Figure 1

RIS, a neuron that inhibits locomotion (Steuer Costa et al., 2019), exhibits increased activity during lethargus (Turek et al., 2013, 2016) or stress-induced sleep (SIS) (Konietzka et al., 2020) as well as during brief episodes of quiescence (Wu et al., 2018). RIS is in the posterior part of the ventral ganglion. We first identified the most posterior neurons in the ganglion, i.e., the paired neurons AVKR and AVKL. We then identified RIS as an unpaired neuron located just anterolateral to AVKR (Video S2). Consistently, in our observation, RIS was active during sleep bouts and exhibited reduced Ca^2+^ activity during motion bouts (Figures 1B and 1C; Video S1).

Video S2. Identification of RIS in AML70 *lite-1(ce314)*; *wtfIs5[Prab-3::NLS::GcaMP6s; Prab-3::NLS::tagRFP]*, related to Figure 1

Among the neurons active during motion bouts, we identified ALA (Figure 1B). ALA is in the dorsal ganglion, which contains four paired neurons, CEPDR, CEPDL, URXR, and URXL, and two unpaired neurons, RID and ALA. We first identified RID, the most anterodorsal neuron, and subsequently identified the two paired neurons and the unpaired neuron ALA (Video S3). ALA activity appeared to largely differ from that of RIS (Figures 1C and S1D; Video S1) and was higher during motion bouts than during sleep bouts across multiple animals (Figure 1D).

Video S3. Identification of ALA in AML70 *lite-1(ce314)*; *wtfIs5[Prab-3::NLS::GcaMP6s*; *Prab-3::NLS::tagRFP]*, related to Figure 1

### ALA activity gradually increases during motion bouts and decreases during sleep bouts

**Figure 2:**
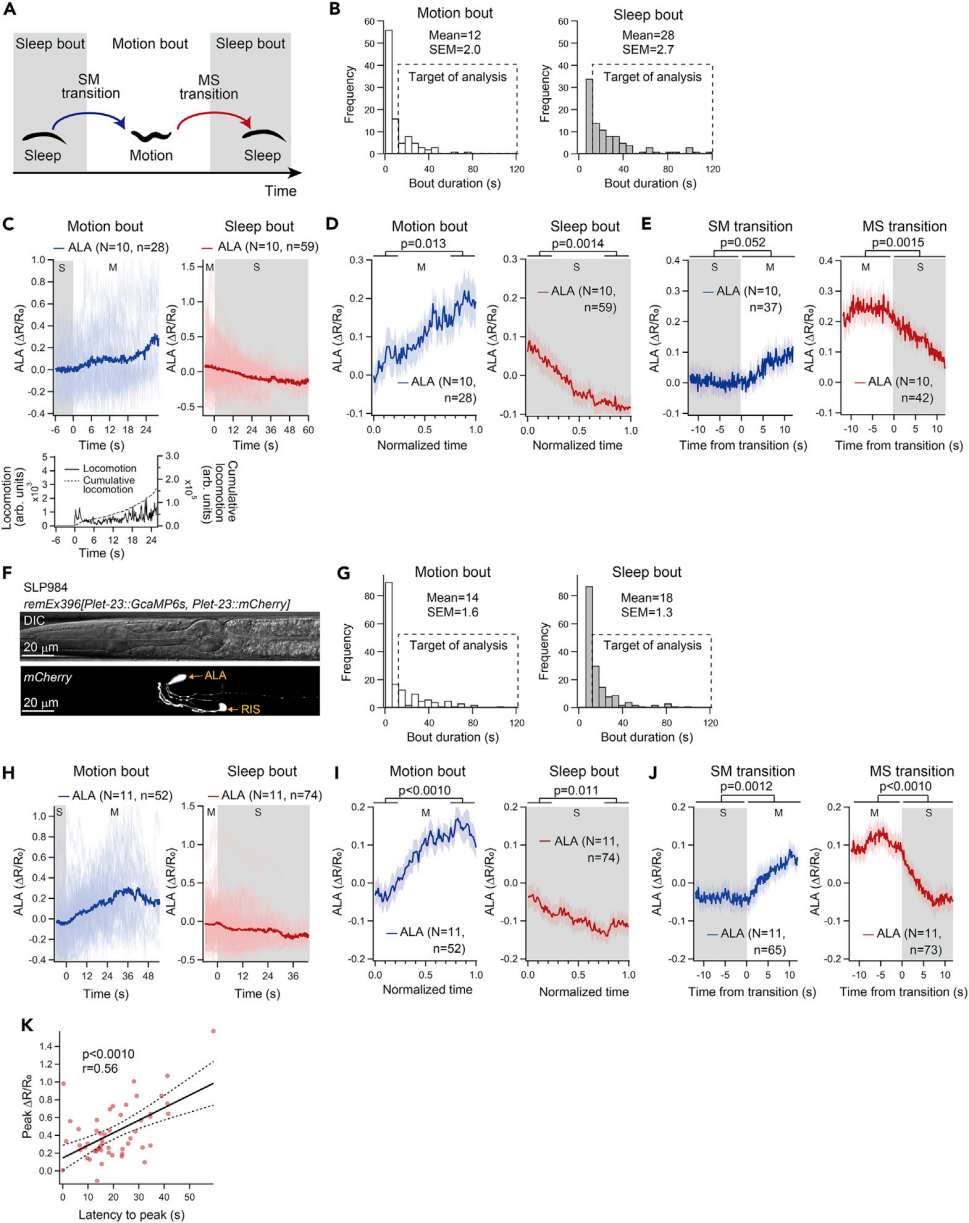
Intracellular Ca^2+^ of ALA gradually increases during motion bouts (A) Schematic of SM and MS transitions. Gray areas represent sleep bouts. (B) The distribution of the durations of motion bouts (left) and sleep bouts (right) in AML70 *lite-1(ce314)*; *wtfIs5[Prab-3::NLS::GcaMP6s; Prab-3::NLS::tagRFP]*. The dashed rectangles indicate the bouts that were targeted in the subsequent ALA activity analyses. (C) Individual (light blue or red) and averaged (dark blue or red) dynamics of ALA intracellular Ca^2+^ during motion bouts (left) or sleep bouts (right) in AML70 *lite-1(ce314)*; *wtfIs5[Prab-3::NLS::GcaMP6s; Prab-3::NLS::tagRFP]*. The locomotor activity is also shown for motion bouts (left lower panel). Gray areas represent sleep bouts. (D) Averaged dynamics of intracellular Ca^2+^ and locomotor activity along motion bouts (left) or sleep bouts (right). Here, the duration of each sleep or motion bout was normalized to 1. Shaded areas indicate ±standard error of the mean (SEM). p-values in Wilcoxon signed-rank test are indicated. (E) Averaged dynamics of Ca^2+^ activity around SM and MS transitions. Shaded areas indicate ±SEM. p-values in paired t test are indicated. Gray areas represent sleep bouts. (F) Expression pattern of *mCherry* in SLP984 *remEx396[*P*let-23::GCaMP6s,* P*let-23::mCherry]*. (G) The distribution of the durations of motion bouts (left) and sleep bouts (right) in SLP984 *remEx396[*P*let-23::GCaMP6s,* P*let-23::mCherry]*. The dashed rectangles indicate the bouts that were targeted in the subsequent ALA activity analyses. (H) Individual (light blue or red) and averaged (dark blue or red) dynamics of intracellular Ca^2+^ during motion bouts (left) and sleep bouts (right) measured in SLP984 *remEx396[*P*let-23::GCaMP6s,* P*let-23::mCherry]*. Gray areas represent sleep bouts. (I) Averaged dynamics of Ca^2+^ activity measured in SLP984 *remEx396[*P*let-23::GCaMP6s,* P*let-23::mCherry]*. Here, the duration of each sleep or motion bout was normalized to 1. Shaded areas indicate ±SEM. p-values in Wilcoxon signed-rank test are indicated. (J) Average dynamics of ALA Ca^2+^ activity around SM and MS transitions in SLP984 *remEx396[*P*let-23::GCaMP6s,* P*let-23::mCherry]*. Shaded areas indicate ±SEM. p-values in Wilcoxon signed-rank test are indicated. Gray areas represent sleep bouts. (K) Correlation between the latency to peak Ca^2+^ activity and the intensity of peak Ca^2+^ activity during motion bouts. Corresponding p-values are the probabilities of obtaining the observed correlation by chance, when the true correlation is zero. The thick dotted line represents the linear regression line and thin dotted lines show the upper and lower limits of the 95% confidential interval for the regression line. N and n in the figure represent numbers of animals and bouts, respectively.

The activation of ALA during motion bouts was unexpected because previous studies reported that ALA is activated during SIS (Konietzka et al., 2020) and that a function of ALA is to promote (or induce) quiescence (Van Buskirk and Sternberg, 2007). Although there were additional neurons that were activated during motion bouts (Figure 1B), ALA was the only neuron that could be reliably identified. Thus, we further analyzed the ALA activity during lethargus in detail. Upon transition from a sleep bout to a motion bout (SM transition), Ca^2+^ activity in ALA gradually increased, and the transition from a motion bout to a sleep bout (MS transition) seemed to occur at the peak of ALA activity followed by decreased ALA activity during the sleep bout (Figures 1C and S1D). We calculated the averaged ALA activity during motion bouts and sleep bouts across multiple animals, focusing on the dynamics of ALA activity around the transitions between the 2 states (Figure 2). In integrating data from multiple bouts, considering that the duration of each motion or sleep bout was highly variable (Figure 2B), bouts shorter than 12 s were omitted. When the raw traces of ALA activity of all motion bouts or sleep bouts were aligned and averaged (Figure 2C; time points where sample numbers were 10 or less were omitted), averaged activity gradually increased during motion bouts, whereas it gradually decreased during sleep bouts. By contrast, the locomotor activity seemed not to gradually increase during the motion bouts, which is in agreement with an essentially linear relationship between locomotor activities and time in the plot of cumulative locomotor activities (Figure 2C). In an alternative analysis, to incorporate data from all time points, the duration of each motion bout or sleep bout was normalized to 1, and the averaged temporal pattern of ALA activity in each type of bout was calculated (Figure 2D). During motion bouts, ALA activity along the normalized time was higher in the fourth quadrant than in the first quadrant (Figure 2D), further supporting that ALA activity gradually increases during motion bouts and decreases during sleep bouts (Figure 2D).

We next analyzed the ALA activity around the transitions between the 2 types of bouts. Average ALA activity during the 12 s before and after SM transitions did not significantly differ (Figure 2E). Conversely, the average ALA activity during the 12 s after MS transitions was lower than that before the MS transition (Figure 2E). Thus, ALA activity did not appear to largely change during SM transitions but decreased during MS transitions. Similar to the Ca^2+^ activity traces during motion bouts and sleep bouts, ALA activity gradually increased following an SM transition, and the MS transition occurred near the peak of ALA activity, followed by a sharp decrease in ALA activity.

We also conducted Ca^2+^ imaging of ALA using a transgenic strain that expresses GCaMP6s under the *let-23* promoter, which is selectively expressed in ALA (Van Buskirk and Sternberg, 2007, 2010). No nuclear localization signals are attached to GCaMP6s in this strain, and GCaMP6s was detected in the cell bodies and neural processes of ALA and RIS (Figure 2F) (Nguyen et al., 2016), and distributions of the duration of motion bouts or sleep bouts were similar to the previous strain (Figure 2G). The results showed an overall similar trend; ALA activity gradually increased during motion bouts, whereas Ca^2+^ activity gradually decreased during sleep bouts (Figures 2H and 2I). Moreover, ALA activity sharply decreased around MS transitions (Figure 2J). The decay of ΔR/R_0_ at the MS transitions appeared to be steeper than that observed in the previous strain (Figures 2E versus 2J); this might be due to the slower kinetics of GCaMP6s in the nucleus compared with the cytosol (Chen et al., 2020; Kim et al., 2014). This observation was further supported by Ca^2+^ imaging of ALA using another transgenic strain that expresses GCaMP6 without any nuclear localization signal under another ALA-selective promoter, the *flp-14* promoter (ZM9078 (Lim et al., 2016); Figure S2E), which also showed a steep decay of ΔR/R_0_ at the MS transitions (Figures S2F, S2G, and S2H).

When we plotted the ALA peak fluorescence signal intensity during each motion bout against the latency to that peak event from the start of the motion bout, we observed a positive correlation (Figure 2K), suggesting that longer motion bouts are accompanied by a higher Ca^2+^ activity peak.

When we observed L4 larvae that are not in lethargus, although ALA exhibited fluctuations in Ca^2+^ activity, an obvious correlation with motion could not be seen (Figure S1C). Ca^2+^ activity of a motor neuron, VA2, was also analyzed during lethargus (Figures S2A-S2D). We used a transgenic strain that expresses GCaMP3 in A-type motor neurons and a few other neurons under the *unc-4* promoter (Gao et al., 2018) (Figure S2A). The patterns of VA2 Ca^2+^ activity were highly variable among motion bouts; in some motion bouts, VA2 exhibited a sudden sharp Ca^2+^ increase, whereas in other bouts, no Ca^2+^ activity was detected (Figure S2B). Therefore, in contrast to ALA, the averaged activity of VA2 did not show an obvious increase during motion bouts (Figures S2C and S2D). Perhaps this is because VA2 is involved in a specific movement that does not necessarily occur in all motion bouts.

### CEH-17 dysfunction leads to abnormal ALA activity and sleep architecture during lethargus

Considering the specific pattern of ALA activity during lethargus, we hypothesized that ALA is involved in MS transitions. We focused on CEH-17, an LIM class homeodomain transcription factor expressed in ALA and SIA neurons (Pujol et al., 2000). Some properties of ALA, including its axon morphology and gene expression patterns, are impaired by a functionally null mutation of *ceh-17* (Pujol et al., 2000; Van Buskirk and Sternberg, 2010).

**Figure 3:**
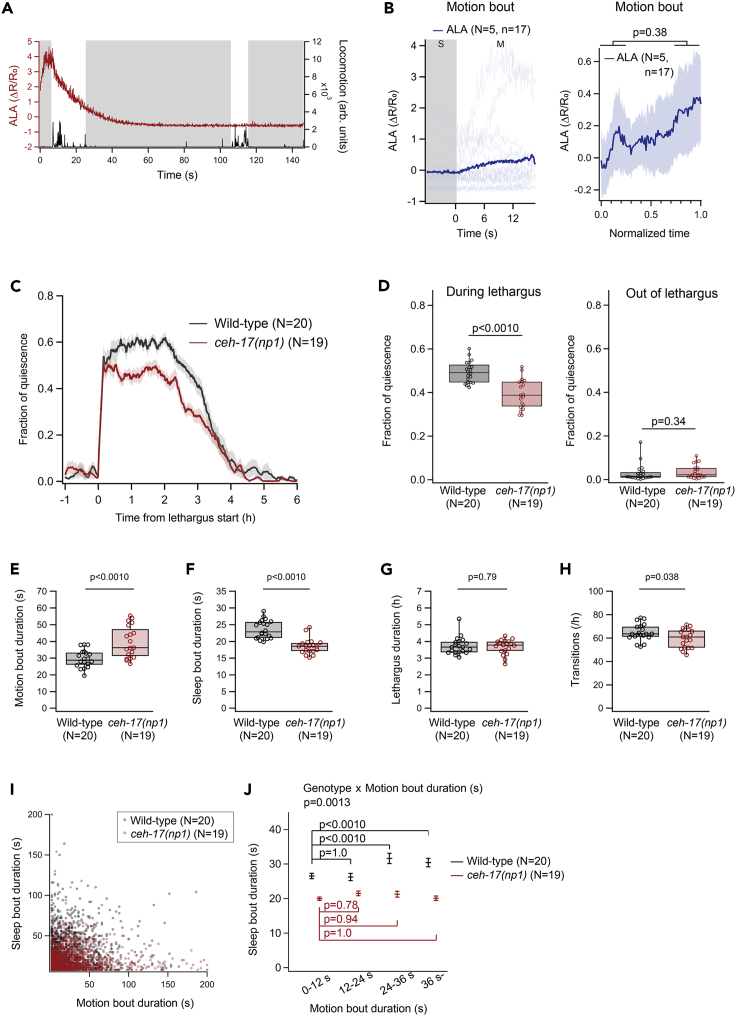
CEH-17 dysfunction leads to abnormal ALA Ca^2+^ dynamics and impaired sleep homeostasis (A) Representative pattern of Ca^2+^ activity (red) and locomotor activity (black) in a *ceh-17(np1)* mutant (SLP930 *ceh-17(np1); lite-1(ce314); wtfIs5[Prab-3:: NLS::GcaMP6s* + *Prab-3::NLS::tagRFP]*). Gray areas represent sleep bouts. (B) Dynamics of ALA Ca^2+^ activity during motion bouts in the *ceh-17(np1)* mutant (SLP930 *ceh-17(np1); lite-1(ce314); wtfIs5[Prab-3:: NLS::GcaMP6s* + *Prab-3::NLS::tagRFP]*). (Left) Individual (light blue) and averaged (dark blue) traces. (Right) Averaged dynamics of Ca^2+^ activity. Here, the duration of each sleep or motion bout was normalized to 1. Shaded areas indicate ±SEM. p-values in Wilcoxon signed-rank test are indicated. (C) Averaged patterns of fraction of quiescence around lethargus in wild-type (black) and *ceh-17(np1)* mutant (red). Shaded areas indicate ±SEM. (D) Comparison of the average fraction of quiescence during lethargus (left) and after lethargus (right). p-values in the t test (left; during lethargus) and the Mann-Whitney’s U test (right; out of lethargus) are indicated. (E–H) Comparison of the average durations of motion bouts (E) and sleep bouts (F), the total length of lethargus (G), and the number of transitions between motion and sleep bouts (H). p-values in the t test (E, F, and H) and the Mann-Whitney’s U test (G) are indicated. (I) Sleep bout duration plotted against the prior motion bout duration in wild-type (black) and *ceh-17(np1)* mutants (red). (J) Comparisons of the distribution of sleep bout durations based on the duration of the prior motion bouts in wild-type (black) and *ceh-17(np1)* mutants (red). Error bars indicate ±SEM. p-value in the interaction between genotype and motion bout duration in two-way ANOVA is indicated above the graph. p-values in the multiple comparison with Sidak correction are indicated in the graph. N and n in the figure represent numbers of animals and bouts, respectively.

In the *ceh-17(np1)* mutant, which is a null mutant (Pujol et al., 2000), ALA activity did not appear to be coupled with motion bouts, i.e., the characteristic pattern of a gradual increase during motion bouts and a decrease during sleep bouts was lost (Figure 3A, 3B, and S3A). There was no significant difference in the average ALA activity between the first and fourth quadrants of the motion bouts (Figure 3B). For reasons unknown, the dynamic range of the ALA calcium imaging data was higher in the mutant (Figure 3A).

We next addressed whether sleep bouts and motion bouts during lethargus were affected in the *ceh-17(np1)* mutant. The behavior of wild-type worms and *ceh-17(np1)* mutants was measured using “artificial dirt” chambers (Lockery et al., 2008) in which worms can freely crawl around, similar to a previous study (Funato et al., 2016). Comparison of the temporal patterns of the fraction of quiescence around lethargus (Figure 3C) and the average fraction of quiescence (Figure 3D) in the *ceh-17(np1)* mutant revealed a reduced fraction of quiescence during lethargus, whereas the activity out of lethargus was unaffected.

Further analyses revealed a prolonged duration of motion bouts (Figure 3E) but a shorter duration of sleep bouts (Figure 3F) in the *ceh-17(np1)* mutant compared with wild-type animals. The total duration of lethargus was not affected (Figure 3G), whereas the total number of transitions between motion and sleep bouts was decreased in the *ceh-17(np1)* mutant (Figure 3H). During lethargus, the duration of a sleep bout positively correlates with the duration of the preceding motion bout, and this correlation is thought to reflect sleep homeostasis (Nagy et al., 2014). When we separated the sleep bouts based on the duration of the preceding motion bouts, in wild-type animals, the duration of sleep bouts following longer motion bouts was longer compared with the duration of those following shorter motion bouts, whereas such tendency was not found in the *ceh-17(np1)* mutant (Figures 3I and 3J).

The involvement of ALA in lethargus is not well established; a laser ablation study demonstrated that ALA-ablated animals exhibit reduced quiescence compared with control animals during lethargus (Van Buskirk and Sternberg, 2007), whereas another study reported that the *ceh-17(np1)* mutant does not exhibit reduced quiescence during lethargus (Katz et al., 2018). Recently, a study showed that some strains in the Caenorhabditis Genetics Center (CGC) carry a nonsense mutation in *fln-2*, termed *ot611*, which affects longevity (Zhao et al., 2019). Given that *fln-2* is expressed in various tissues (Demaso et al., 2011) and that longevity and sleep interrelate, we addressed the genotype of *fln-2* in the *ceh-17* mutant strains used in this study. We found by single nucleotide polymerase chain reaction (Gaudet et al., 2009) that IB16, which is the *ceh-17(np1)* mutant strain stocked in the Caenorhabditis Genetics Center, carries the *fln-2(ot611)* allele. We therefore compared the lethargus phenotype between wild-type, *ceh-17(np1)*, and IB16 *ceh-17(np1); fln-2(ot611)*. The results demonstrated that the decreased average fraction of quiescence in the *ceh-17(np1)* single mutant was suppressed in IB16 *ceh-17(np1); fln-2(ot611)* (Figures S3B and S3C). Interestingly, although the reduced average fraction of quiescence was suppressed in IB16 *ceh-17(np1); fln-2(ot611)*, similar to the *ceh-17(np1)* single mutant, no significant difference was detected in the duration between sleep bouts that followed either longer or shorter motion bouts in IB16 *ceh-17(np1); fln-2(ot611)* (Figures S3D and S3E), suggesting that the *fln-2(ot611)* mutation (or another unidentified mutation in IB16 *ceh-17(np1); fln-2(ot611)*) increased the quiescence but did not suppress the impaired sleep homeostasis caused by CEH-17 dysfunction.

### Optogenetic activation of ALA during motion bouts causes premature transitions to sleep bouts

**Figure 4:**
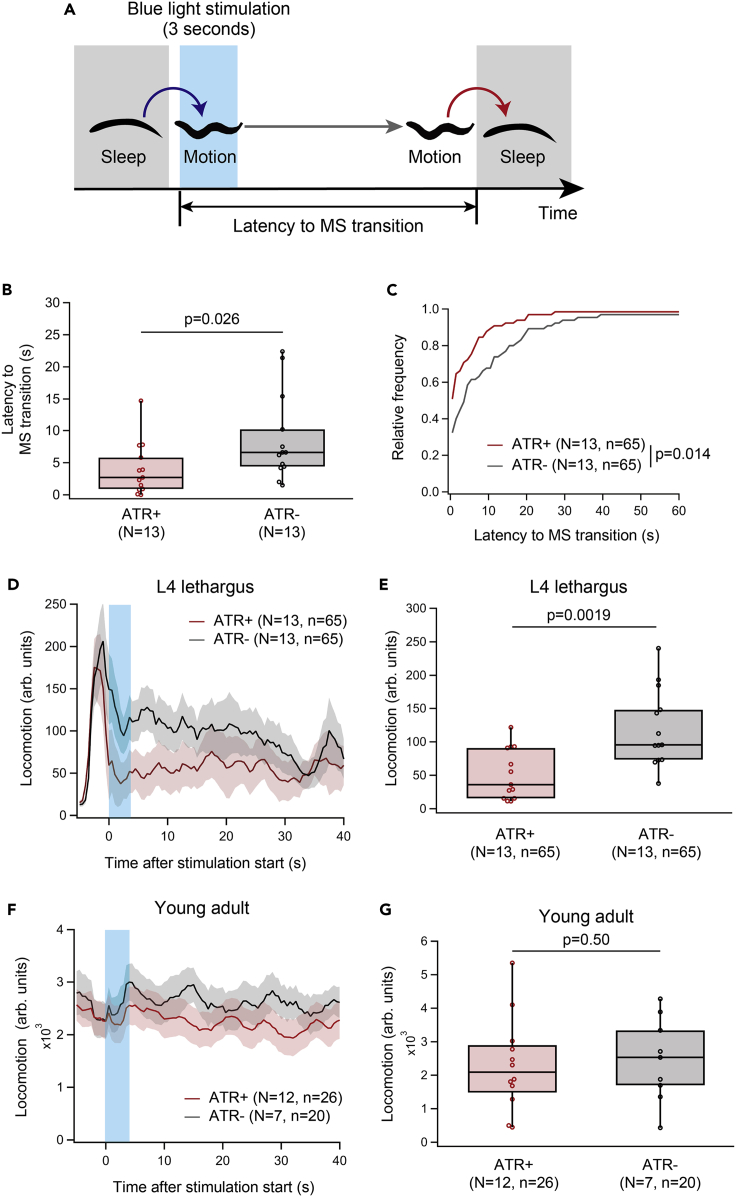
Optogenetic activation of ALA during motion bouts promotes transitions to sleep bouts (A) Schematic of the experiment. Blue light stimulation was applied for 3 s upon transition to a motion bout. (B) Comparison of the latency to MS transitions following light stimulation. p-value in Mann-Whitney’s U test is indicated. (C) Comparison of the cumulative distribution of the latency to MS transitions following light stimulation. p-value in the Kolmogorov-Smirnov test is indicated. (D–G) Comparison of the pattern of locomotor activity around the light stimulation (D, F) and the averaged locomotor activity during the 5 s after light stimulation (E and G) between ATR+ and ATR− worms in lethargus. Shaded areas indicate ±SEM. p-values in unpaired t test are indicated. N and n in the figure represent numbers of animals and trials, respectively.

To directly examine the effects of ALA activation, we applied optogenetics to artificially activate ALA in worms that move freely on agar plates. The light-gated cation channel channelrhodopsin-2 (C128S) fused to GFP (ChR2(C128S):GFP) was expressed under the *ver-3* promotor, which is expressed exclusively in ALA in the nervous system (Popovici et al., 2002; Van Buskirk and Sternberg, 2007, 2010) (Figure S4A). In the experimental group, the animals were cultivated on all-*trans*-retinal (ATR) containing plates, which is essential for the light-mediated activation of ChR2 (ATR + group). Control animals were cultivated without ATR (ATR-group). The animals were exposed to blue light for 3 s at the onset of the motion bouts during lethargus (Figure 4A).

The duration of motion bouts with blue light stimulation was decreased in the ATR + group compared with the ATR-group, as revealed by comparison of the average durations (Figure 4B) or cumulative distributions (Figure 4C), indicating that optogenetic activation of ALA prematurely terminates motion bouts. This was further supported by quantifying locomotor activity just after the light stimulation (Figure 4D). Comparison of the average locomotor activity for 10 s after the blue light stimulation revealed decreased average locomotor activity in the ATR + group compared with the ATR-group (Figure 4E). When the same experiment was conducted in awake adult worms, optogenetic activation of ALA did not significantly affect locomotion (u), suggesting that the function of ALA differs among behavioral states or ages.

## Discussion

In the present study, we found that Ca^2+^ activity gradually increases in the interneuron ALA during motion bouts and decreases during sleep bouts in lethargus. ALA activation promotes transitions from motion bouts to sleep bouts, as revealed by optogenetics experiments. The characteristic activity pattern of ALA requires normal function of the homeodomain transcription factor CEH-17, which is expressed selectively in ALA. Worms deficient in *ceh-17* exhibited prolonged motion bouts and shortened sleep bouts. These findings demonstrate that ALA and CEH-17 play crucial roles in homeostatic sleep regulation, which is required to shape the sleep architecture during lethargus.

Homeostatic regulation is a critical feature of sleep. Yet, the underlying mechanisms are largely unknown. Homeostatic sleep regulation is predicted to require a system that holds information on the preceding amount of wakefulness. On the basis of our findings, ALA seems to be involved in this system in nematodes; the intracellular Ca^2+^ concentration in ALA gradually increases during active bouts and moreover peak ALA activity positively correlates with the duration of the motion bout. Similarly, the intracellular Ca^2+^ concentration of a subset of neurons in the ellipsoid body of fruit flies gradually increases during prolonged wakefulness (Liu et al., 2016). In both cases, how the amount of preceding wakefulness is reflected in the levels of intracellular Ca^2+^ remains unknown.

To the best of our knowledge, there are no reports of neurons in mammals that exhibit such characteristic changes in intracellular Ca^2+^ levels, although reports on the patterns of Fos expression in some sleep-promoting neurons in the hypothalamus and brainstem (Kashiwagi et al., 2020; Liu et al., 2017) imply that the intracellular Ca^2+^ levels in these neurons also reflect prior amounts of wakefulness.

Further studies are needed to uncover how ALA works as a homeostatic regulator of sleep. ALA receives synaptic inputs from the multimodal sensory neurons ADE and ADL (Ailion and Thomas, 2000; Bargmann et al., 1993; Jang et al., 2012; Li et al., 2011; Sambongi et al., 1999). ALA might somehow accumulate information regarding the sensory input to these neurons. Another possibility is that the increase in the intracellular Ca^2+^ in ALA is caused by mechanical sensory input, as ALA itself is a high-threshold mechanosensory neuron (Sanders et al., 2013). Considering that ALA Ca^2+^ levels closely matched the prior amount of locomotion, mechanosensory or other types of sensory input generated by locomotion might cause the gradual increase in intracellular Ca^2+^. An as-yet unidentified neuron lying upstream of ALA might also be generating a similar activity pattern and transmitting it to ALA.

Optogenetic activation of ALA during motion bouts promoted transitions to sleep bouts. Thus, increased levels of intracellular Ca^2+^ in ALA during natural sleep likely contribute to transitions from motion bouts to sleep bouts. Notably, a *ceh-17(np1)* mutant strain (IB16) is reported to exhibit normal levels of quiescence during lethargus (Katz et al., 2018), implying that ALA is not involved in regulating lethargus. We found, however, that this mutant strain carried a mutation in *fln-2*, and outcrossing the strain resulted in significantly reduced quiescence during lethargus. Thus, a mutation in *fln-2* or some other gene likely masked the phenotype of the *ceh-17(np1)* mutant. Importantly, homeostatic regulation of sleep bouts was impaired in the *ceh-17(np1)* mutant regardless of the strain, providing further support that ALA is involved in sleep homeostasis. ALA has direct input to AVA, ADE, AVE, and RMD (White et al., 1986). ALA is also connected with RID via a gap junction (White et al., 1986). Of the downstream neurons, AVE is a command interneuron that controls backward locomotor activity. ALA and AVE are involved in generating short bouts of quiescence that are occasionally observed outside of lethargus (Katz et al., 2018). Thus, inhibition of AVE by ALA might contribute to inhibiting locomotion at the transitions to sleep bouts during lethargus. RIS, a neuron that strongly inhibits locomotion (Steuer Costa et al., 2019), might also act downstream of ALA. During stress-induced sleep, activation of ALA precedes the activation of RIS (Konietzka et al., 2020). We observed a similar trend during lethargus. Although there are no direct synaptic connections between ALA and RIS, ALA may activate RIS indirectly or via paracrine secretion of neurotransmitters or neuromodulators.

The molecular mechanisms that underlie the characteristic gradual increase of the intracellular Ca^2+^ concentration in ALA or ellipsoid body neurons in the fruit fly during wakefulness remain to be elucidated. Many studies in mammals have attempted to identify extracellular molecules that accumulate during wakefulness and promote sleep pressure. Adenosine is a well-known somnolent, and according to microdialysis studies, its extracellular concentration in the basal forebrain increases following prolonged wakefulness (Porkka-Heiskanen et al., 1997). Monitoring the extracellular adenosine levels in the basal forebrain using a recently developed G-protein-coupled receptor-activation based sensor, however, did not result in a positive correlation between the preceding amount of wakefulness and adenosine levels (Peng et al., 2020). Other than extracellular molecules, the phosphorylation of various synaptic proteins is enhanced by prolonged wakefulness (Vyazovskiy et al., 2008; Wang et al., 2018). Such modulation of synaptic proteins might lead to changes in the intracellular Ca^2+^ levels and the excitability of neurons that are engaged in homeostatic sleep regulation.

Accumulating evidence supports that the proteins involved in sleep are highly conserved among the animal phyla. For example, the AP2 transcription factor APTF-1 was identified as a factor required for specification of the sleep-promoting neuron RIS in nematodes, and subsequent studies revealed that AP2 transcription factors also play crucial roles in regulating sleep in fruit flies and mice (Hu et al., 2020; Kucherenko et al., 2016; Nakai et al., 2020). The homeodomain transcript factor CEH-17 is essential for the differentiation of ALA, and based on our findings, it is possible that CEH-17 homologues have conserved roles in regulating sleep homeostasis. Further analyses of CEH-17 homologues may help to identify molecular pathways and neurons involved in sleep homeostasis in mammals.

### Limitation of the study

We were not able to confirm the identity of many neurons in the Ca^2+^ imaging. Accurate identification of each neuron and evaluation of its activity are necessary to reveal the complete picture of the circuit mechanisms that underlie the characteristic patterns of ALA activity. Once the molecular and circuit mechanisms that generate the characteristic activity patterns of ALA are uncovered and hence the basis of sleep homeostasis are elucidated in *C. elegans*, the obvious and essential question is whether such mechanisms are utilized by other animal species including mammals.

## STAR★Methods

### Key resources table

**Table:** 

REAGENT or RESOURCE	SOURCE	IDENTIFIER
**Bacterial and virus strains**		

*Escherichia coli*: strain OP50-1	CGC	OP50-1 https://cgc.umn.edu/strain/OP50-1

**Chemicals, peptides, and recombinant proteins**		

all-trans-retinal	Sigma-Aldrich	Cat# R2500-25MG

**Deposited data**		

Original data	Mendeley Data	https://doi.org/10.17632/sp25w5kxvd.1

**Experimental models: Organisms/strains**		

wild-type strain	Caenorhabditis Genetics Center (CGC)	N2 https://cgc.umn.edu/strain/N2
*ceh-17(np1) Ⅰ; fln-2(ot611) X*	CGC	IB16 https://cgc.umn.edu/strain/IB16
*ceh-17(np1) Ⅰ*	This study	SLP769
*ceh-14(ch3) X; fln-2(ot611) X*	CGC	TB528 https://cgc.umn.edu/strain/TB528
*lite-1(ce314) X*; *wtfIs5[Prab-3::NLS::GcaMP6s; Prab-3::NLS::tagRFP]*	(Hallinen et al., 2021)	AML70 https://cgc.umn.edu/strain/AML70
*hpIs459[Punc-4-GCaMP6s::mCherry* + *lin-15(* + *)]*	(Gao et al., 2018)	ZM8428 https://cgc.umn.edu/strain/ZM8428
*hpIs587 [*P*flp-14::GcaMP6::mCherry, lin-15(* + *)]*	(Lim et al., 2016)	ZM9078 https://cgc.umn.edu/strain/ZM9078
*ceh-17(np1) Ⅰ; lite-1(ce314) X; wtfIs5[Prab-3:: NLS::GcaMP6s* + *Prab-3::NLS::tagRFP]*	This study	SLP930
*remEx394[Pver-3::ChR2(C128S)::GFP]*	This study	SLP983
*remEx396[Plet-23::GcaMP6s, Plet-23::mCherry]*	This study	SLP984

**Recombinant DNA**		

*Prab-3::NLS::GCaMP6s, Prab-3::NLS::GCaMP6s*	Addgene	RRID:Addgene_68119; http://n2t.net/addgene:68119
pJH2447	Gift from Mei Zhen	pJH2447
pDONR201	Gift from Yuichi Iino	pDONR201
pPD-DEST	Gift from Yuichi Iino	pPD-DEST
pENTR*-*P*ver-3*	This study	pYH343
pENTR*-*P*let-23*	This study	pYH344
pENTR*-*P*ceh-17*	This study	pYH251
pPD-DEST*-GCaMP6s*	This study	pYh340
pPD-DEST-*mCherry*	This study	pYH12
pPD-DEST*-ChR2(C128S)::GFP*	This study	pYH110
P*ver-3::ChR2(C128S)::GFP*	This study	pYH348
P*let-23::GCaMP6s*	This study	pYH346
P*let-23::mCherry*	This study	pYH350
P*ceh-17::ChR2(C128S)::GFP*	This study	pYH342

**Software and algorithms**		

Python (version 3.9)		RRID: SCR_008394; https://www.python.org/
Igor Pro 8	WaveMetrics, USA	RRID:SCR_000325; https://www.wavemetrics.com/
SPSS (version 28.0)	IBM, USA	RRID:SCR_019096; https://www.ibm.com/products/spss-statistics
Fiji	NIH, USA	RRID:SCR_002285; https://fiji.sc/
Script for Ca^2+^ imaging analysis	This study; GitHub	https://github.com/hayashi-laboratory/Worm_Ca_imaging
Script for behavioral analysis	This study; GitHub	https://github.com/hayashi-laboratory/Worm_behavior_analysis
Script for optogenetics	This study; GitHub	https://github.com/hayashi-laboratory/Worm_optogenetics

### Resource availability

#### Lead contact

Further information and requests for resources and reagents should be directed to and will be fulfilled by the lead contact, Yu Hayashi (yuhayashi@g.ecc.u-tokyo.ac.jp).

#### Materials availabilityƒ

*C. elegans* strains, plasmids, or other reagents are available from the Lead contact upon reasonable request.

### Experimental model and subject details

#### Strains and culture condition

All the strains used in this study are listed in the Key resource table. Standard culture conditions were used (Brenner, 1974). Worms were cultured at 20°C on Nematode Growth Medium (NGM) plates that were seeded with *Escherichia coli* OP50-1. The experiments were performed at 20°C ± 1°C.

### Method details

#### fln-2 genotyping and backcross

All animals used in this study were genotyped with respect to fln-2 by allele-specific polymerase chain reaction as previously described (Gaudet et al., 2009; Zhao et al., 2019). Backcross with wild-type animals was conducted to eliminate the fln-2(ot611) mutation.

#### Plasmid DNA construction and transformation

All expression plasmids were generated by Gateway system (Invitrogen, ThermoFisher, USA) as previously reported (Matsuki et al., 2006). To generate pENTR plasmids with promotor sequences, the promotor regions of let-23 and ver-3 were amplified with PCR from genomic DNA and ligated into the pDONR201. The following primers were used for amplification:

Plet-23 5′-GTACAAAAAAGCAGGCTAGTTGGTGAGAGTGACAAAA-3′ &

5′-GTACAAGAAAGCTGGGTGCCTCCCAGAAAATTGTAGA-3′,

Pver-3 5′-GTACAAAAAAGCAGGCTCCACTGCCACGTCATATTCA-3′ &

5′-GTACAAGAAAGCTGGGTTGAGCTTCAATTTCATCTCAGAA-3′

To generate pDEST plasmids with GCaMP6s and unc-54 3′ UTR, the coding sequence of GCaMP6s was amplified from Prab-3:NLS:GCaMP6s. Prab-3:NLS:GCaMP6s was a kind gift from Andrew Leifer (Addgene plasmid # 68119; http://n2t.net/addgene:68119; RRID:Addgene_68119). The amplified sequence was ligated into pPD-DEST with an In-Fusion Cloning Kit (Clontech, TaKaRa Bio, Japan). The sequence of ChR2(C128S) was cut from pJH2447, which was a kind gift from Mei Zhen. The fragments were also ligated into pPD-DEST.

The expression constructs containing the promotor, gene, and 3′ UTR were constructed with an LR recombination reaction between the pENTR plasmids and pDEST plasmids.

The transgenic strains were produced by the germline transformation method as previously described (Mello et al., 1991). The expression constructs were injected with 10 ng/μL pRF4 containing *rol-6(su1006)* as a transformation marker and pUC18 as a carrier DNA.

#### Ca^2+^ imaging with Worm Sheet

A confocal imaging microscope system (LSM800, Carl Zeiss, Germany) was utilized for Ca^2+^ imaging. For excitation, a 488- and 561-nm laser was used for GCaMP6s and RFP, respectively. Fluorescence emission signals were separated by a variable secondary dichroic (VSD) beamsplitter, and those below 600 nm were passed through an SP545 emission filter to be detected as GCaMP-derived signals, and those above 600 nm as RFP-derived signals. GCaMP- and RFP-derived signals were detected with gallium arsenide phosphide photomultiplier tubes (GaAsP-PMT). Differential interference contrast (DIC) images were obtained using a transmitted light detector (T-PMT). All signals were detected simultaneously.

Animals that entered lethargus within 1 h were mounted in commercially available custom-made microfluidic chambers (Worm Sheet, Biocosm Inc, Japan). Worm Sheet used in this study is an ultra-thin PDMS microfluidic chamber containing linear microfluidic channels 18 μm deep and 20 μm wide. We loaded worms in 3 μL of M9 buffer in one of the channels and covered it with cover glass.

Single plane imaging was conducted using a 40x objective lens, and 512x56 pixel images were acquired at 10 frames per second. The plane containing ALA was selected as the imaging plane. The time required to acquire one plane was 60 ms. The number of neurons imaged varied from 4 to 41 depending on the angle of the worm. A total of 2000 images were obtained during an imaging session, except for the recording in Figures 1B and 1C, in which approximately 2400 images were obtained at 6.67 frames per second.

Image analysis was conducted by Fiji (NIH, USA, RRID:SCR_002285) (Schindelin et al., 2012) and custom-made Python scripts. All fluorescent images were loaded in Fiji. All nuclei or cell bodies were detected and tracked with Trackmate (Tinevez et al., 2017). The difference of Gaussian filter was used for nuclei detection and simple LAP tracker. After tracking nuclei or cell bodies, the mean fluorescent intensity data was calculated. Both GCaMP6s and RFP intensities were then used to calculate the ratio. Ca^2+^ activity was estimated according to the fractional change of the ratio, (R-R_0_)/R_0_, where R_0_ is defined as the mean of R.

Obtained DIC images were analyzed with the image subtraction method using custom-made Python scripts. Under our conditions, if a pixel in a subtracted image derived from 2 sequential images had a pixel value higher than the threshold, the pixel was counted as different between the 2 sequential images. To define the threshold, 2000 subtracted images were obtained from a microfluidic chamber that does not contain a worm imaged under the same condition, and the mean plus 5 standard deviations of the pixel values was set as the threshold. If the number of pixels that were different exceeded 100, the worm was judged as locomotor active at that time point. Based on this criterion, the state of the worm at each time point was assigned either as locomotor active or non-locomotor active. Next, each time point was further classified to either a sleep bout or a motion bout based on the following criteria; a sleep bout was defined as a series of time points classified as non-locomotor active that lasts longer than 6 s. The remaining series of time points were classified as motion bouts. For analyses of the Ca^2+^ imaging data, bouts shorter than 12 s were excluded. For calculating average GCaMP6s signal dynamics within each bout type and correlations (Figures 2B, 2C, 2G, 2H, and 2J), bouts at the beginning or end of the imaging sessions were excluded.

To paralyze L4 larvae, the animals were loaded to M9 buffer containing 10 mM sodium azide. The fraction of quiescence was defined as the ratio of time points in which worms were non-locomotor active to the total time in 1.5-min time windows.

For the analyses of correlation between the ALA peak fluorescence signal intensity and the latency to that peak event from the start of the motion bouts (related to Figure 2K), traces of ALA fluorescence signal intensity were smoothened by calculating a 1 s moving average.

#### Behavioral analysis using artificial dirt chambers

Similar to a previous report (Funato et al., 2016), lethargus and related behavioral measurements were recorded using “artificial dirt” chambers (Lockery et al., 2008) filled with OP50-1 containing liquid NGM. Behavior was recorded using a stereomicroscope (SZX16, OLYMPUS, Japan) equipped with a CCD camera (GR500BCM2, Shodensha, Japan). The animals were imaged at 1 frame per 2 s at 40x magnification.

Behavioral data were analyzed with the custom-made Python scripts. Lethargus was defined and analyzed following a previous report with some modifications (Funato et al., 2016). Briefly, the time-lapse images were processed with the image subtraction script and the locomotor activity data was extracted. If the locomotor activity at each time-point was below 1% of the body size, the worm was judged as quiescent at that time. The fraction of quiescence was then calculated in 10-min time windows. Similar to the Ca^2+^ imaging data analyses, a sleep bout was defined as a series of time points classified as non-locomotor active that lasts longer than 6 s, and the remaining series of time points were classified as motion bouts.

#### Optogenetics experiments

The animals were cultured on NGM with or without 100 μM all-trans-retinal (Sigma Aldrich, USA). The plates were stored in the dark. NGM plates for the assay were freshly seeded 1 day before the experiment.

Late L4 worms were observed every 30 min, and when pharyngeal pumping ceased for more than 30 s, the worm was judged to be in lethargus and moved to the assay plate set under an upright stereomicroscope (SZX16, Olympus, Japan) at 40x magnification. The experiment was conducted under dim light and the eye lens was covered to avoid excessive light. Light stimuli with a duration of 3 s and intensity of 0.22 mW/mm^2^ was provided using an LED ring light (CCS Inc., Japan) with a center wavelength of 470 nm.

Time-lapse imaging was performed at 2 frames per second. Upon setting a worm under the stereomicroscope, baseline behavior was recorded for at least 10 min. Subsequently, light was shined onto the plate when the animal entered a motion bout. A minimum interval of 5 min was set when performing the next light exposure experiment, and the light exposure experiment was repeated at most 5 times for each animal. For light exposure experiments using adult animals, because adult animals are constantly active, light exposure was initiated immediately after the baseline recording, and the experiment was repeated at most 3 times per animal.

Custom-made python scripts were used for analyzing the optogenetic experiment data. Following image subtraction, locomotor quiescent states lasting for more 5 s were categorized as sleep bouts. Motion bout duration time (related to Figures 4B and 4C) was defined as the duration of the motion bout after the light stimulation. For the analyses of the effect of light stimulation on locomotion (related to Figures 4D–4G), traces of locomotion were smoothened by calculating a 2.5 s moving average.

### Quantification and statistical analysis

#### Statistical analysis

All statistical analysis was conducted with SPSS (IBM, USA, RRID:SCR_019096), and all the figures were drawn by Igor Pro 8 (WaveMetrics, USA, RRID:SCR_000325). Sample sizes, statistical tests, and *P*-values are indicated in the figure or figure legends. Where applicable, all statistical tests were 2-tailed. p-values were considered statistically significant when less than 0.05. In the boxplots, the box represents the 25–75% range, and the horizontal line represents the median. Whiskers represent ±2σ.

## Data Availability

All raw data analyzed in this paper are available at Mendeley (Mendeley Data: https://doi.org/10.17632/sp25w5kxvd.1). The python scripts for Ca^2+^ imaging analysis (https://github.com/hayashi-laboratory/Worm_Ca_imaging), lethargus behavior analysis (https://github.com/hayashi-laboratory/Worm_behavior_analysis), and optogenetics (https://github.com/hayashi-laboratory/Worm_optogenetics) are available at GitHub. Any additional information required to reanalyze the data reported in this paper is available from the Lead contact upon request.
