# Supplementary material for: Intracellular Ca2+ dynamics in the ALA neuron reflect sleep pressure and regulate sleep in Caenorhabditis elegans
Source: iScience. 2022 May 23;25(6):104452. doi: 10.1016/j.isci.2022.104452 (PMC9189131; doi:10.1016/j.isci.2022.104452)
Supplement: Document S1. Figures S1–S4 [file mmc1.pdf]

iScience, Volume *25*

Supplemental information

Intracellular Ca^{2+} dynamics in the ALA neuron reflect sleep pressure and regulate sleep in *Caenorhabditis elegans*

Shinichi Miyazaki, Taizo Kawano, Masashi Yanagisawa, and Yu Hayashi

SUPPLEMENTAL INFORMATION

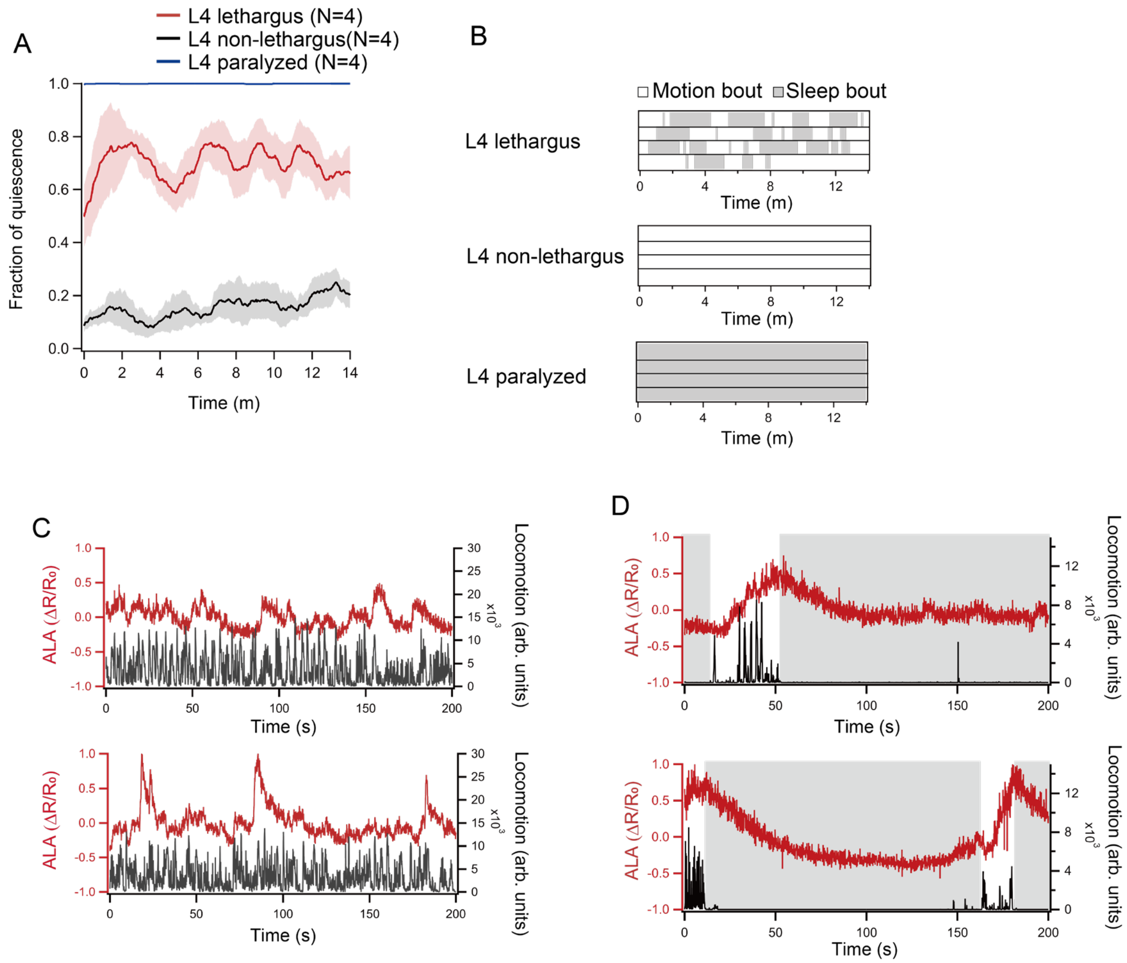

Figure S1. Detailed analyses of the behavior and ALA intracellular Ca^{2+} dynamics in AML70 *lite-1(ce314)*; *wtfIs5[Prab-3::NLS::GcaMP6s; Prab-3::NLS::tagRFP]* (related to Figures 1 and 2)

(A, B) Comparison of the fraction of quiescence (A) and the representative patterns of sleep bouts and motion bouts (B) among L4 lethargus, non-lethargus, and paralyzed larvae. Shaded areas in (A) indicate ±SEM . N in the figure represent numbers of animals.

(C) Representative patterns of ALA Ca^{2+} activity (red) and locomotor activity (black) in L4 non-lethargus larvae.

(D) Additional representative patterns of ALA Ca^{2+} activity (red) and locomotor activity (black) in L4 lethargus larvae. Grey areas represent sleep bouts.

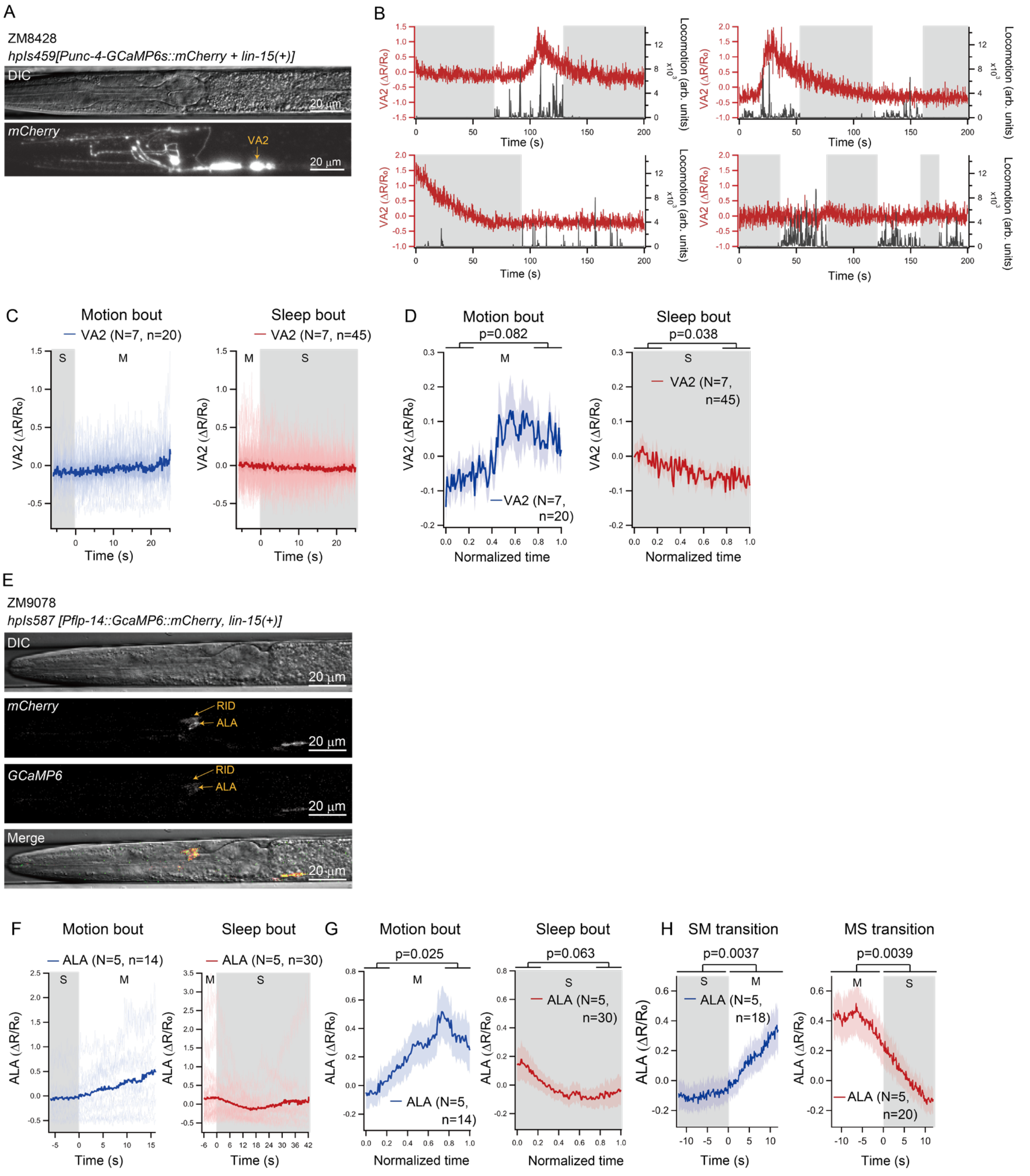

Figure S2. Ca^{2+} imaging of the VA2 and ALA (related to Figures 2 and 3)

(A) Expression pattern of *mCherry* in ZM8428 *hpIs459 [unc-4p::GCaMP3::mCherry + lin-15(+)]*.

(B) Representative patterns of VA2 intracellular Ca^{2+} activity (red) and locomotor activity (black). Grey areas represent sleep bouts.

(C) Individual (light blue or red) and averaged (dark blue or red) dynamics of VA2 intracellular Ca^{2+} during

motion bouts (left) or sleep bouts (right). Grey areas represent sleep bouts.

(D) Averaged dynamics of intracellular Ca^{2+} and locomotor activity along motion bouts (left) or sleep bouts (right). Here, the duration of each sleep or motion bout was normalized to 1. Shaded areas indicate ±SEM. P-values in paired t-test (motion bout) and Wilcoxon signed-rank test (sleep bout) are indicated.

(E) Expression patterns of *mCherry* (middle) and *GCaMP6* (lower) in ZM9078 *hpIs587 [*P*flp-14::GcaMP6::mCherry, lin-15(+)].*

(F) Individual (light blue or red) and averaged (dark blue or red) dynamics of ALA intracellular Ca^{2+} during motion bouts (left) or sleep bouts (right) in ZM9078 *hpIs587 [*P*flp-14::GcaMP6::mCherry, lin-15(+)]*. Grey areas represent sleep bouts.

(G) Averaged dynamics of intracellular Ca^{2+} and locomotor activity along motion bouts (left) or sleep bouts (right). Here, the duration of each sleep or motion bout was normalized to 1. Shaded areas indicate ±SEM. P-values in paired t-test (motion bout) and Wilcoxon signed-rank test (sleep bout) are indicated.

(H) Averaged dynamics of Ca^{2+} activity around SM and MS transitions. Shaded areas indicate ±SEM. P-values in paired t-test are indicated. Grey areas represent sleep bouts.

N and n in the figure represent numbers of animals and bouts, respectively.

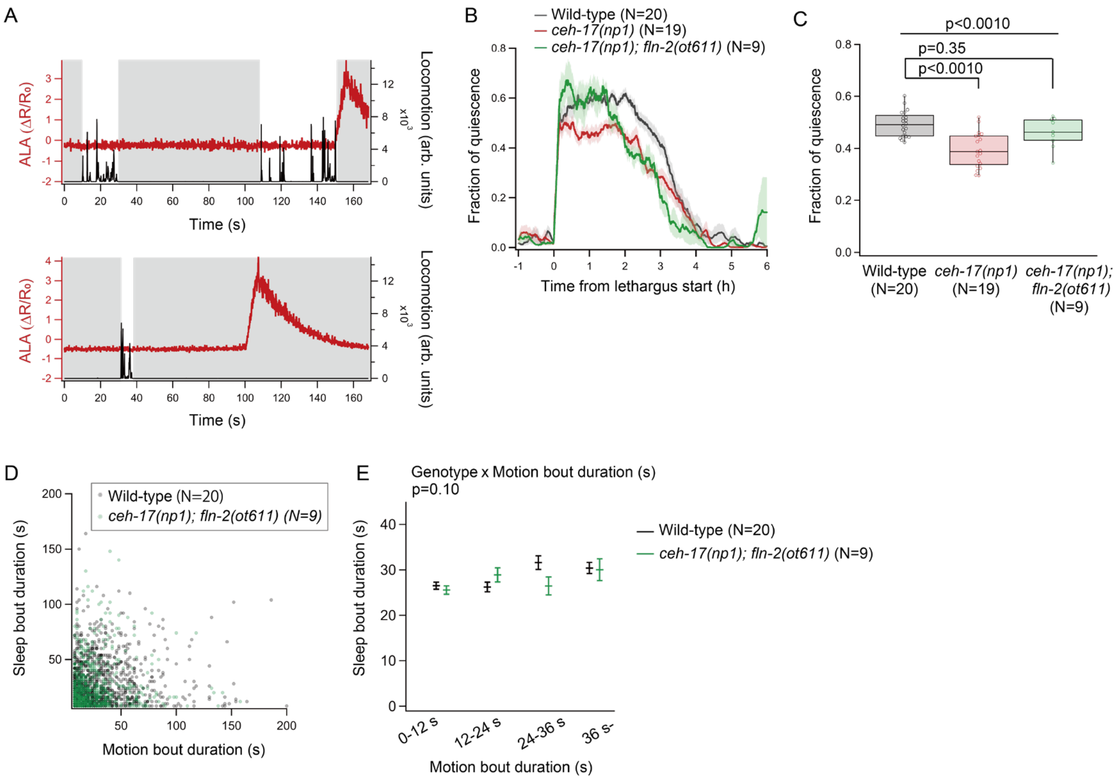

Figure S3. Detailed analyses of strains carrying *ceh-17(np1)* (related to Figure 3)

(A) Additional representative patterns of Ca^{2+} activity (red) and locomotor activity (black) in the *ceh-17(np1)* mutant (SLP930 *ceh-17(np1); lite-1(ce314); wtfIs5[Prab-3:: NLS::GcaMP6s + Prab-3::NLS::tagRFP]*). Grey areas represent sleep bouts.

(B) Averaged patterns of fraction of quiescence around lethargus in wild-type (black), SLP769 *ceh-17(np1)* mutant (red), and IB16 *ceh-17(np1); fln-2(ot611)* mutant (green). The same data as in Figure 3C are shown for wild-type and SLP769. Shaded areas indicate ±SEM.

(C) Comparison of the mean fraction of quiescence among wild-type, SLP769 *ceh-17(np1)*, and IB16 *ceh-17(np1); fln-2(ot611)*. The same data as in Figure 3D are shown for wild-type and SLP769. P-values in the Tukey multiple comparison test are indicated.

(D) Sleep bout duration plotted against the prior motion bout duration in wild-type (black) and IB16 *ceh-17(np1); fln-2(ot611)* mutants (green). The same data as in Figure 3I are shown for wild-type.

(E) Comparisons of the distribution of sleep bout durations based on the duration of the prior motion bouts in wild-type (black) and IB16 *ceh-17(np1); fln-2(ot611)* mutants (green). The same data as in Figure 3J are shown for wild-type. P-value in the interaction between genotype and motion bout duration in two-way ANOVA is indicated above the graph.

N and n in the figure represent numbers of animals and bouts, respectively.

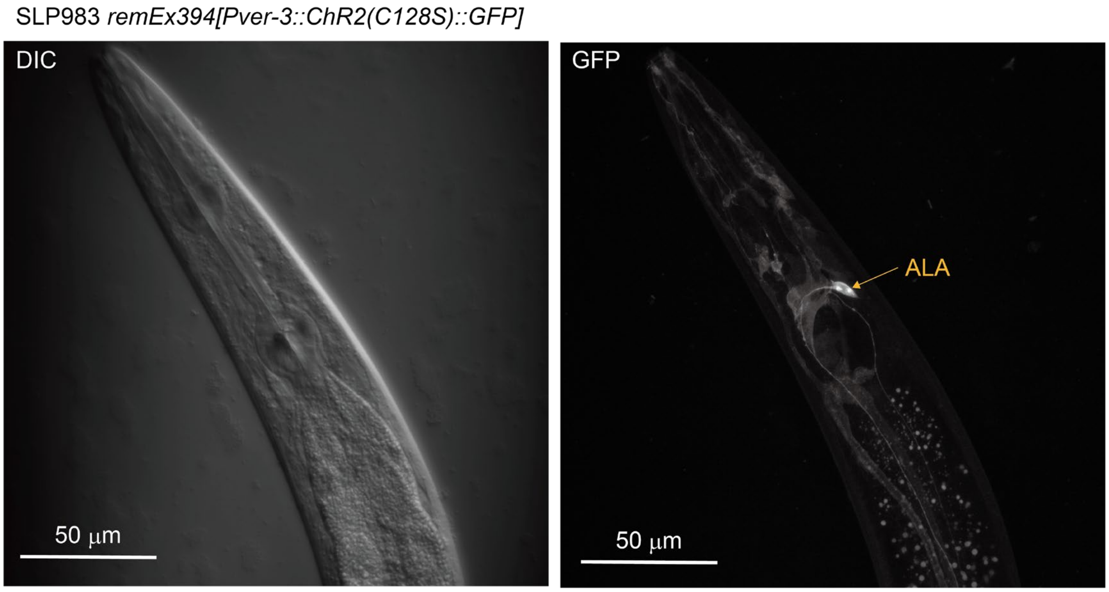

Figure S4. Expression pattern of ChR2(C128S)::GFP in SLP983 *remEx394[*P*ver-3::ChR2(C128S)::GFP]* (related to Figure 4)

Representative confocal image of SLP983 *remEx394[*P*ver-3::ChR2(C128S)::GFP]*.

DIC image (left) and fluorescent image (right) are shown. Arrow indicates the ALA cell body.
